# Implications of m6A methylation and microbiota interaction in non-small cell lung cancer: From basics to therapeutics

**DOI:** 10.3389/fcimb.2022.972655

**Published:** 2022-09-02

**Authors:** Fen-Sheng Qiu, Jia-Qi He, Yu-Sen Zhong, Mei-Ying Guo, Chen-Huan Yu

**Affiliations:** ^1^ Key Laboratory of Experimental Animal and Safety Evaluation, Zhejiang Academy of Medical Sciences (Hangzhou Medical College), Hangzhou, China; ^2^ Pharmaceutical Department, The First Affiliated Hospital of Zhejiang Chinese Medical University, Hangzhou, China; ^3^ Cancer Hospital of the University of Chinese Academy of Sciences (Zhejiang Cancer Hospital), Hangzhou, China; ^4^ Institute of Basic Medicine and Cancer, Chinese Academy of Sciences, Hangzhou, China

**Keywords:** epigenetics, biomarker, METTL3, FTO, inhibitor, m6A

## Abstract

N6-methyladenine (m6A) is one of the most common RNA epigenetic modifications in all higher eukaryotes. Increasing evidence demonstrated that m6A-related proteins, acted as oncogenes or tumor suppressors, are abnormally expressed in the cell lines and tissues of non-small cell lung cancer (NSCLC). In addition, lung as the special immune organ contacts with the outer environments and thereby inevitably suffers from different types of microbial pathogen attack. Those microbial pathogens affect the development, progression, and clinical outcomes of NSCLC *via* altering host m6A modification to disrupt pulmonary immune homeostasis and increase the susceptibility; conversely, host cells modulate m6A modification to repress bacterial colonization. Therefore, m6A harbors the potential to be the novel biomarkers and targets for predicting poor prognosis and chemotherapy sensitivity of patients with lung cancer. In this paper, we provided an overview of the biological properties of m6A-modifying enzymes, and the mechanistic links among lung microbiota, m6A modification and NSCLC. Although the flood of novel m6A-related inhibitors represents many dramatic improvements in NSCLC therapy, their efficacy and toxicity in NSCLC are explored to address these pivotal gaps in the field.

## Introduction

Epigenetic modifications are heritable changes in gene expression caused without altering the DNA nucleotide sequence and the development of tumors. In recent years, with the continuous development of tumor epigenetic research, especially following the in-depth study of abnormal DNA methylation, microRNA and non-coding RNA dysregulation and histone modification, N6-methyladenosine (m6A) has not only ushered in a new era of post-transcriptional gene regulation in eukaryotes, but also rapidly become a hot spot in the field of RNA methylation modification (Zhang et al., 2020). It is a hot topic of research in RNA methylation modification. As the most prevalent internal mRNA modification in all higher eukaryotes, m6A has been shown to be aberrantly expressed in a variety of tumors and plays an important role in the regulation of cell proliferation, invasion, metastasis and other malignant biological behaviors (Chen et al., 2021; Xu et al., 2021; Zhang et al., 2021; Deng et al., 2022).

Microbiota disorders or microbial pathogens in the lungs have been identified to affect the occurrence, development, and prognosis of non-small cell lung cancer (NSCLC) through different means, such as infllammation, metabolism, and cell signaling transduction (Shi et al., 2021). Moreover, some common risk factors like COPD may play a significant role on the populations of pulmonary microbiota in patients with NSCLC (He et al., 2022). However, the molecular mechanism of microbiota in the prognosis of NSCLC remains poorly elucidated.

This paper reviews the research progress on m6A and non-small cell lung cancer induced by microbiota, aiming to provide a theoretical basis for a profound understanding of non-small cell lung carcinogenesis and the search for tumor predictive biomarkers and therapeutic targets.

## Biological properties of m6A

m6A, a methylation occurring at the adenosine N6 position, was first detected in poly(A) RNA in 1974 and was thought to be a potentially broad modification with the potential to selectively control gene expression (Desrosiers et al., 1974). However, the technical means of detection at that time could not meet the requirements for the study of the biological significance of m6A modifications. With the development of immunoprecipitation techniques as well as high-throughput sequencing, which only enabled researchers to target m6A modifications with greater precision, m6A regained the focus of attention and opened a new chapter in RNA epigenetic modifications. Recent studies have revealed that m6A is a very common modification in mRNA and ncRNA that affects RNA shear, translation, stability, and the epigenetic effects of certain ncRNAs, containing on average 1 to 2 m6A residues per 1000 nucleotides. m6A occurs mainly in the 3′ noncoding region, the stop codon, and in the RRACH sequence near the long exon (where R=A or G and H=A, C, or U) (Tang et al., 2021; Deng et al., 2022). There is growing evidence that m6A affects almost all stages of mRNA metabolism, from processing in the nucleus to translation and decay in the cytoplasm, which in turn affects circadian rhythms, regulates the cell cycle, accelerates cell state changes, regulates cell differentiation and reprogramming, and ultimately affects homeostasis of the body, causing a variety of diseases, including tumors (Zaccara et al., 2019; Gao et al., 2021; Kumari et al., 2021).

**Figure 1:**
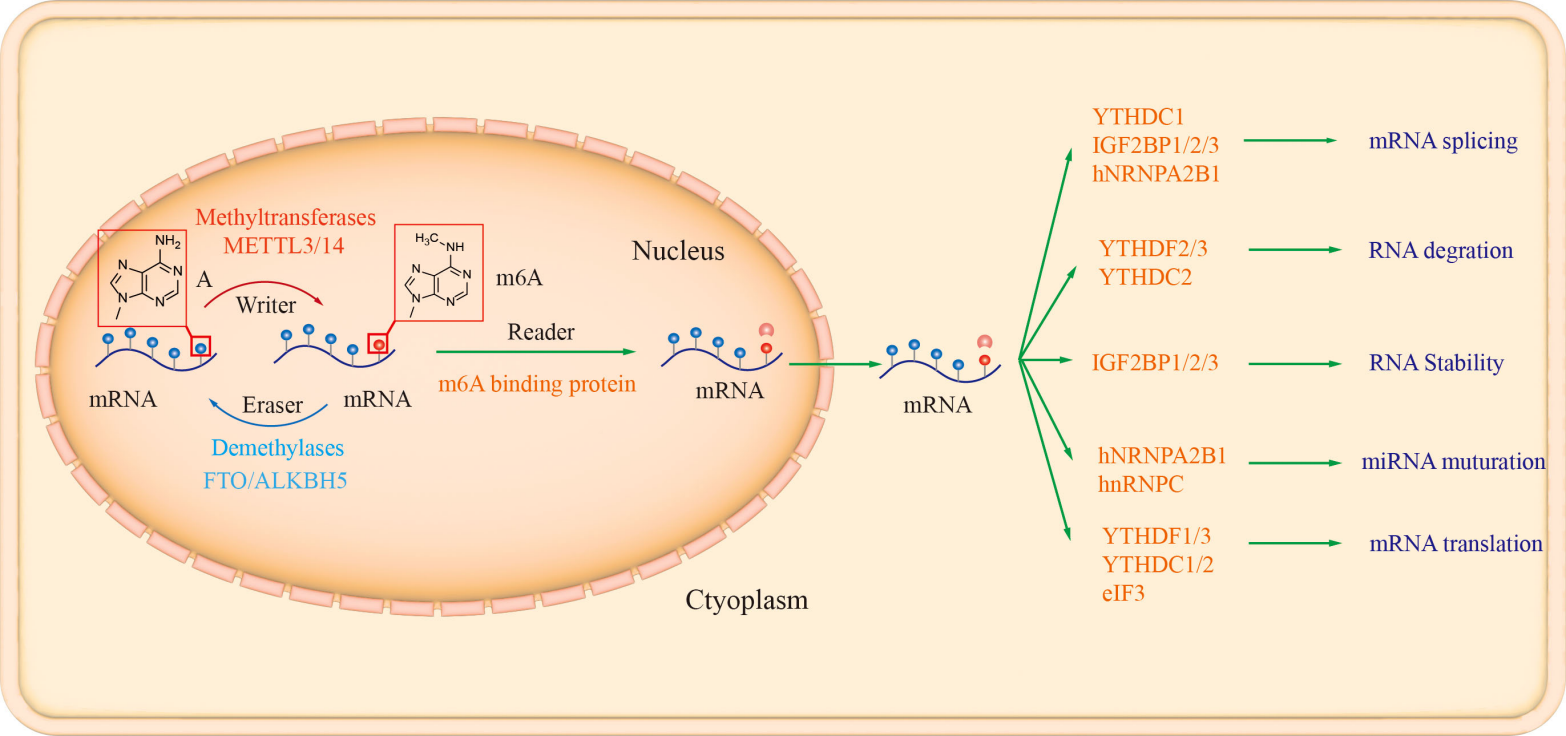
The biology functions of m^6^A enzymes. There are three different types of m6A enzymes, namely “Writer” (such as METTL3, METTL14, WTAP, and KIAA1429), “Eraser” (ALKBH5 and FTO), and “Reader” (YTHDF1/2/3, YTHDC1/2, YTHDC2 HNRNPA2B1, HNRNPC and IGF2BP1/2/3, which involved in mRNA degradation, translation, stability, and non-coding RNA processing (Tang et al., 2021; Deng et al., 2022).

Similar to DNA and histone methylation, RNA m6A methylation modification is a dynamic and reversible epistatic regulatory process, mainly regulated by methylesterases (writers), demethylases (erasers) and recognition proteins (readers). The m6A methyltransferases, such as methyltransferase like 3/14 (METTL3/14), Wilm’s tumor 1-associated protein (WTAP), and the demethylases FTO and AlkB homolog 5 (AlkB homolog 5, ALKBH5), are responsible for the methylation and demethylation of intracellular mRNAs, respectively. In contrast, recognition proteins such as YT521-B homology (YTH) family proteins YTHDF1-3, nuclear members YTHDC1-2, and heterogeneous nuclear ribonucleoproteins (hnRNPs), recognize m6A modifications of mRNAs and thus regulate mRNA biological behaviors (**Figure 1**).

## The roles of m6A-RNA methylation in NSCLC

The RNA epigenetic modification system is complex, and m6A modifying enzymes can recognize both oncogenes and anti-oncogenes. Previous studies have shown that m6A-RNA methylation plays an important role in the development of NSCLC, and its methylation-related factors can act as oncogenes (e.g., ALKBH5) or anti-oncogenes (e.g., METTL3, FTO, YTHDF1, HNRNPA2B1, HNRNPC and eIF3, etc.) which play roles in NSCLC cell proliferation, migration, invasion, apoptosis and cell cycle, and may be potential new targets for NSCLC therapy.

### m6A methylation transferases in NSCLC

The m6A modification is catalyzed by the methyltransferase complex (Writers) consisting of methyltransferase-like 3 (METTL3) and methyltransferase-like 14 (METTL14) and its WTAP, RBM15, KIAA1429, ZC3H13, and METTL16. Among them, METTL3 is the catalytic subunit active, METTL14 can form a dimer with METTL3, and WTAP recruits METTL3-METTL14 dimer to nucleosites for methylation modification of the substrate by binding to RBM15, KIAA1429, and ZC3H13 to form a complex (Wang et al., 2016). METTL3 is the major catalase in the m6A methyltransferase system, which can act as a proto-oncogene or anti-oncogene to participate in biological processes such as tumorigenesis, proliferation, invasion, migration, cell cycle, and differentiation (Lin et al., 2016; Zeng et al., 2020; Shi et al., 2022). Currently, the role of m6A methyltransferases in NSCLC is mainly focused on METTL3-related studies. It has been shown that knockdown of METTL3 can inhibit the proliferation, drug resistance and metastasis of NSCLC cells and induce apoptosis, as well as alter the phosphorylation of PI3K/AKT signaling pathway members, thereby exerting oncogenic effects (Jin et al., 2021; Zhang et al., 2021). The alternation of m6A in mRNAs mediated by SUMO-ization modification of METTL3 and the subsequent alteration of gene expression profiles may directly affect lung cancer H1299 cell growth (Du et al., 2018). In addition, METTL3 may also affect NSCLC progression in a non-m6A-dependent manner. METTL3 enhances translation efficiency and promotes NSCLC metastasis by interacting with eIF3h, which cyclizes mRNA and increases the efficiency of ribosome recycling and reuse. Inhibition of METTL3 may not only be a target for the treatment of NSCLC, but also enhance chemotherapy sensitivity (Jin et al., 2021). Those findings suggested that METTL3 could play a predominantly pro-cancer role in NSCLC cells and be a potentially diagnostic and therapeutic target for NSCLC.

### m6A demethylases in NSCLC

The main m6A demethylases are fass obesity-associated protein (FTO) and alkane hydroxylase homolog 5 (ALKBH5), both of which belong to the ALKB homolog family and are classified as 2-oxoglutarate and iron-dependent nucleic acid oxygenases (Wang et al., 2020). FTO and ALKBH5 primarily demethylate m6A- modified bases, with FTO being the first m6A demethylase identified, showing high demethylation activity for m6A, and ALKBH5 catalyzing the removal of m6A modifications from nuclear RNAs (mainly mRNA), thereby affecting metabolic disease and human obesity. FTO was the first m6A demethylase to be identified, showing high demethylation activity against m6A, which can affect metabolic diseases and the development of obesity in humans, while ALKBH5 can catalyze the removal of m6A modifications from nuclear RNA (mainly mRNA), which in turn can affect nuclear RNA export, metabolism and gene expression, and even fertility in mice (Zheng et al., 2013; Huang et al., 2020; Prakash et al., 2021). However, the current studies showed that although ALKBH5 and FTO are both m6A demethylases, they play different roles in the pathological development of NSCLC. Among them, ALKBH5 mainly plays an oncogenic role in NSCLC, while FTO may play a pro-cancer role in NSCLC. It was found that aberrantly expressed ALKBH5 down-regulated Yes-associated protein (YAP) expression mediated by YTHDFs and inhibited miR-107/LATS2-mediated YAP activity, thereby inhibiting NSCLC cell proliferation, invasion, migration and epithelial mesenchymal transition (Jin et al., 2020). Knockdown of the FTO gene in lung squamous cell carcinoma cells was found to effectively inhibit cell proliferation and promote apoptosis, while overexpression of FTO promoted their malignant progression (Liu et al., 2018). Overexpression of FTO in lung adenocarcinoma cells also showed enhanced proliferation, migration and invasion, and downregulation of m6A-RNA expression, indicating that FTO may promote lung adenocarcinoma progression through m6A demethylation leading to cell viability, migration, and invasion *in vitro* (Wang et al., 2021). Moreover, FTO was overexpressed and m6A content was sharply reduced in NSCLC tissues and cell lines. Further knockdown of FTO expression in cells was found to inhibit NSCLC cell proliferation, and the mechanism of action may be related to the demethylation enzyme activity of FTO (Li et al., 2019). Meanwhile, it was found that FTO overexpression did not promote NSCLC cell proliferation and invasion after mutation, suggesting that the oncogenic effect of FTO on NSCLC may mainly depend on its catalytic activity (Liu et al., 2018). The above findings suggest that FTO plays a pro-carcinogenic role in NSCLC and inhibits FTO control may slow the progression of NSCLC.

### m6A methylated reading protein in NSCLC

Emerging studies have shown that m6A methylated reading proteins mainly act as pro-cancer factors affecting NSCLC proliferation, migration and invasion. There are m6A reading proteins in organisms that specifically recognize and bind to m6A and mediate its exercise of biological functions, including YTHDF1, YTHDF2, YTHDC1, hnRNPA2B1 and hnRNPC, eIF3, IGF2BP1, etc. These proteins bind specifically to m6A to mediate selective shearing, intracellular localization and translational control of RNA metabolic processes (Huang et al., 2018; Shi et al., 2019; Hu et al., 2021).

YTHDF1 deficiency could affect the translation efficiency of CDK2, CDK4 and cyclin D1 and inhibit NSCLC cell proliferation and lung squamous cell carcinoma progression, while high YTHDF1 expression was associated with better clinical outcome (Shi et al., 2019). Compared with para-cancerous tissues, hnRNPA2B1 was highly expressed in NSCLC and could bind to ERK/p53/HDM2 signaling pathway, suggesting that hnRNPA2B1 may be involved in NSCLC pathogenesis (Kim et al., 2021; Jin et al., 2022). In addition, hnRNPC overexpression is associated with later clinical stage and lymph node and distant metastasis, and promotes NSCLC cell proliferation, migration and invasion, possibly through activation of the IFN-α-JAK-STAT1 signaling pathway (Yan et al., 2019).

**Figure 2:**
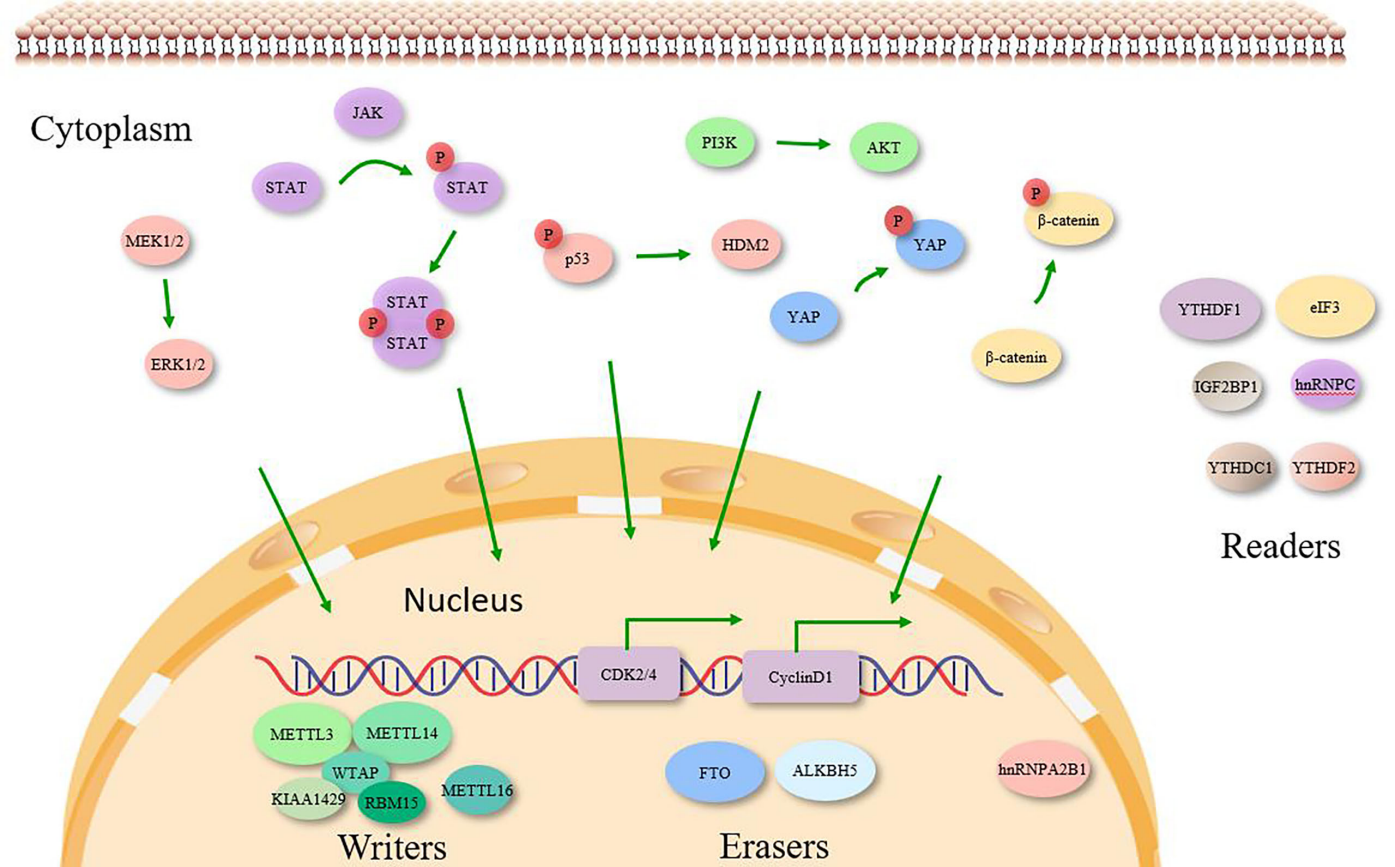
Molecular targets of m6A enzymes in various signaling pathways. m6A ‘Writers’, ‘Erasers’ and ‘Readers’ play significant role in cancer by targeting specific RNA transcripts of cell signaling molecules causing activation/inactivation of various intracellular signaling pathways, which are indicated by the colors corresponding to the regulators.

eIF3 is the largest and most complex translation initiation factor. eIF3 is one of the largest and most complex translation initiation factors, consisting of 13 subunits, such as eIF3a, eIF3b, eIF3d and eIF3h. Among them, eIF3a is the housekeeping gene, which is closely associated with lung carcinogenesis and drug resistance (Yin et al., 2018). In NSCLC cells, eIF3b was highly expressed and was associated with disease progression and poor prognosis, and also promoted NSCLC cell proliferation and inhibited apoptosis. Knockdown of eIF3d in NSCLC cells was also found to significantly inhibit cell proliferation and colony formation, and to block the cell cycle in G2/M phase. This effect may be achieved by inhibiting integrin α 5 and TNFRSF21 expression or activating the β-linked protein signaling pathway (Desnoyers et al., 2015; Lin et al., 2015). In addition, direct physical and functional interactions between eIF3h and METTL3 have been reported to be required for enhanced translation of oncogenic mRNAs, formation of densely packed polyribosomes and oncogenic transformation of lung adenocarcinoma (Choe et al., 2018). In contrast, one study reported that eIF3h protein was highly expressed in lung adenocarcinoma tissues and that eIF3h overexpression promoted lung adenocarcinoma cell migration and invasion, which further confirmed that eIF3h is an oncogenic factor in lung adenocarcinoma (Esteves et al., 2020). Thus, it can be seen that eIF3 subunits in NSCLC mainly exhibit proliferation-promoting, migration, invasion and apoptosis-inhibiting effects on cancer cells, and are expected to be potential targets for NSCLC therapy (**Figure 2**).

### m6A enzymes have potential to be new diagnostic biomarkers and therapeutic target for NSCLC

It was well known that m6A is one of the most common internal mRNA modifications in higher eukaryotic. Notably, accumulating evidences have demonstrated that m6A enzymes are widely involved in various biological process of NSCLC, including proliferation, metastasis as well as inflammatory response (**Table 1**). Thus, m6A enzymes may provide us effectively diagnostic biomarkers for molecular diagnosis of NSCLC and efficient therapeutic intervention targets for NSCLC treatment. It has been reported that METTL3 with high expression level was closely associated with shorter overall survival, which indicated that METTL3 may have potential to be a prognostic biomarker in NSCLC (Shi et al., 2021). Additionally, leukocyte m6A was not only positively related to the number of lymphocytes but also negatively correlated with monocytes in NSCLC, which was mainly caused by upregulated METTL3 and downregulated FTO and ALKBH5. Therefore, leukocyte m6A and m6A enzymes may be novel noninvasive biomarkers for NSCLC diagnosis (Pei et al., 2020). Moreover, Shen et al. have suggested that eIF3a played an essential role in radically resected NSCLC patients and it was of prognostic value for aberrant eIF3a expression to independently predict prognosis of NSCLC (Shen et al., 2014). Consequently, these findings indicated that m6A enzymes, as m6A RNA methylation regulators, have potential to be used as diagnostic and prognostic biomarkers, suggesting great clinical value.

**Table 1:** The roles of m6A enzymes in NSCLC progression.

Type	m6A enzymes	Regulation	Role in NSCLC	Reference
Writers	METTL3	Up-regulation	Induces NSCLC drug resistance and metastasis	Jin et al., 2019
	METTL14	Down-regulation	Suppresses the malignant progression of NSCLC	Yang et al., 2021
Erasers	FTO	Up-regulation	Accelerates tumor growth and metastasis	Wang et al., 2021
	ALKBH5	Up-regulation	Inhibits tumor growth and metastasis	Jin et al., 2020
Readers	YTHDF1	Down-regulation	represses NSCLC cell proliferation, invasion and migration abilities, whereas enhances apoptosis	Zhou et al., 2020
	eIF3	Up-regulation	inhibits proliferation and cisplatin sensibility in NSCLC	Fang et al., 2017

## m6A inhibitors

The main representative drugs are azacitidine, vorinostat and other specific inhibitors of epigenetic- related proteins, which play a significant role in clinical practice. Given that epigenetic inheritance is based on the molecular mechanism of regulation of transcription and post-transcriptional products, and that the results of previous studies have shown that m6A plays an important role in the malignant biological behavior of tumors, the development of specific inhibitors of m6A-related proteins is of great scientific significance and clinical value (**Table 2**).

**Table 2:** The inhibitors of m6A-related enzymes and their biological function.

Inhibitor	IC50 (mM)	Target	Biological function	Reference
Rhein	21	FTO	Inhibit FTO by competitively binding the catalytic domain against ssRNA substrate, also effectively inhibit m6A demethylation *in vitro* and increase cellular levels of m6A	Chen et al., 2021
MA	17.4	FTO	Bind and stabilize FTO but had minimal influence on ALKBH5	Huang et al., 2015
Radicicol	16.0	FTO	Radicicol, as an FTO inhibitor *in vitro*, provided new information on designing more potent compounds to inhibit the activity of the enzyme	Wang et al., 2018
N-CDPCB	4.95	FTO	Inhibitory activity on FTO demethylation of the 15-mer ssRNA, significantly decreased the level of m6A of mRNA in preadipocytes	He et al., 2015
CHTB	39.2	FTO	CHTB complexed with human FTO reveals that the novel small molecule binds to FTO in a specific manner, regulation of mRNA splicing and adipogenesis by modulating m6A levels	He et al., 2015
Entacapone	3.5	FTO	Entacapone as a chemical inhibitor of FTO mediating metabolic regulation through FOXO1	Peng et al., 2019
IOX3	2.8	FTO/ALKBH5	An inhibitor of the HIF prolyl hydroxylases, decreased cellular protein expression of FTO, failed to alter the m6A level inside of cells. IOX3 also could bind to ALKBH5 in a covalent attachment	Han et al., 2019
FMN	Unknown	Nucleoside	Combined with blue-light irradiation substantially decreases m6A levels in cells by directly targeting the nucleoside modification	Xie et al., 2018
Clausine E	Unknown	FTO	Bound by positive entropy and negative enthalpy changes.	Wang et al., 2019
6-chloro-2-phenyl-1H-benzimidazole(CPBZD)	24.65	FTO	Bound by positive entropy.	Li et al., 2019

### FTO inhibitors

The development of inhibitors based on m6A-related enzymes has focused on the first discovered RNA demethylase, FTO, a conserved class of 2OG oxygenases, whose activity can be inhibited by the universal inhibitor NOG. Rhein, a natural anthraquinone in the Rhubarb, can inhibit FTO by competitively binding to single- stranded RNA (ssRNA) substrate in the catalytic domain, which can inhibit the demethylation activity of FTO on m6A on mRNA *in vitro* and *in vivo*, thereby increasing the level of m6A in cells (Chen et al., 2021). Meclofenamic acid (MA), a selective FTO inhibitor, could bind to and stabilize FTO without affecting the demethylation enzymatic activity of ALKBH5, and could significantly increase the level of cellular m6A, but had no significant effect on the level of m6A in FTO-deficient cells, indicating that the crystal structure of the FTO/MA complex clearly demonstrates that β3i and β4i in FTO form hydrophobic pockets that specifically recognize MA, while ALKBH5 lacks the corresponding structure, providing a chemical basis for the development of specific FTO inhibitors (Huang et al., 2015).

Aik et al. identified a series of compounds that inhibit FTO activity by competing with 2OG through metal ion chelating groups, but such inhibitors need to be developed to avoid inhibition of other 2OG oxygenases and to improve specificity.the compounds identified by Zheng et al. effectively inhibited FTO of their 2OG oxygenase family and had IC50 values close to those of the broad- spectrum 2OG enzyme inhibitor NOG (Aik et al., 2013). Based on the molecular mechanism of FTO recognition of m6A modified substrates and other characteristics, Huang et al. applied crystal structure-based compound design and synthesis optimization to obtain FTO small molecule inhibitors. The compounds selectively inhibited the demethylation of FTO in AML cells, upregulated the m6A modification on the mRNA of key AML genes, increased the abundance of oncogenic proteins such as ASB2 and RARA, and decreased the abundance of oncogenic proteins such as MYC and CEBPA, thereby inhibiting the proliferation of AML cells, and demonstrated the anti-leukemia therapeutic effect in a PDX mouse model. This study indicates a new direction for molecularly targeted intervention of m6A modification to affect gene expression for anti-tumor research (Huang et al., 2019).

Huang et al.also found that entacapone, which was previously approved by the FDA for marketing, could act as a specific inhibitor of FTO based on a virtual screening of the structure and a series of *in vitro* and *in vivo* bioactivity assays. It directly binds and inhibits FTO activity *in vitro*. In a diet-induced obesity mouse model, feeding entacapone with FOXO1 mRNA as a direct-acting substrate for FTO induced intrahepatic glycogen xenobiogenesis and adipose tissue thermogenesis, resulting in reduced body mass and fasting glucose concentrations in mice (Peng et al., 2019). In addition, FTO is not only closely related to obesity and tumors, but its common variants rs9939609 may be associated with central nervous system diseases such as brain volume loss and alcohol dependence (Qiao et al., 2016; Liu et al., 2018). Therefore, the potential use of FTO inhibitors, in addition to anti-tumor and weight loss, may also be developed as drugs for neurological diseases.

### Other m6A-related protein inhibitors and chemical interventions

Both ALKBH5 and FTO belong to the ALKB subfamily of the Fe(II)/2-oxoglutarate (2OG) dioxygenase superfamily. 2OG dioxygenase superfamily members act on a variety of substrates and are involved in the regulation of protein biosynthesis. IOX3 can also bind covalently to ALKBH5. Under ALKBH5 crystallization conditions, citrate competes with 2OGs and Mn(ii) at the active site of alkanes and can be directed towards the development and modification of ALKHB5 as an inhibitor (Xu et al., 2014; Han et al., 2019). Among methylesterases, METTL3 is the main active catalytic site, while METTL14 plays a key role in the substrate recognition process (Wang et al., 2016). Due to the limited knowledge of the mechanism of methylesterase recognition and catalytic RNA methylation, the development of its inhibitors has been relatively slow. So far, only 3-deazaadenosine (DAA) has been found to inhibit METTL3, but the effect of DAA is broad-spectrum, inhibiting the activity of all RNA methylesterases, without specificity for m6A methylesterases.

In addition, chemical demethylation of m6A is another important chemical intervention strategy. Riboflavin was used as the exogenous photosensitive molecule riboflavin to selectively and efficiently oxidize m6A of mRNA catalyzed by LED blue light, thus reducing the m6A modification of mRNA (Xie et al., 2017). They further developed flavin mononucleotide (FMN), which can perform m6A modification removal of mRNA on living cells, can chemically demethylate m6A in cells catalyzed by LED blue light, thus realizing mRNA m6A-specific demethylation in living cells using the compound.

## m6A modification regulates progression of cancer mediated by microbiota

**Figure 3:**
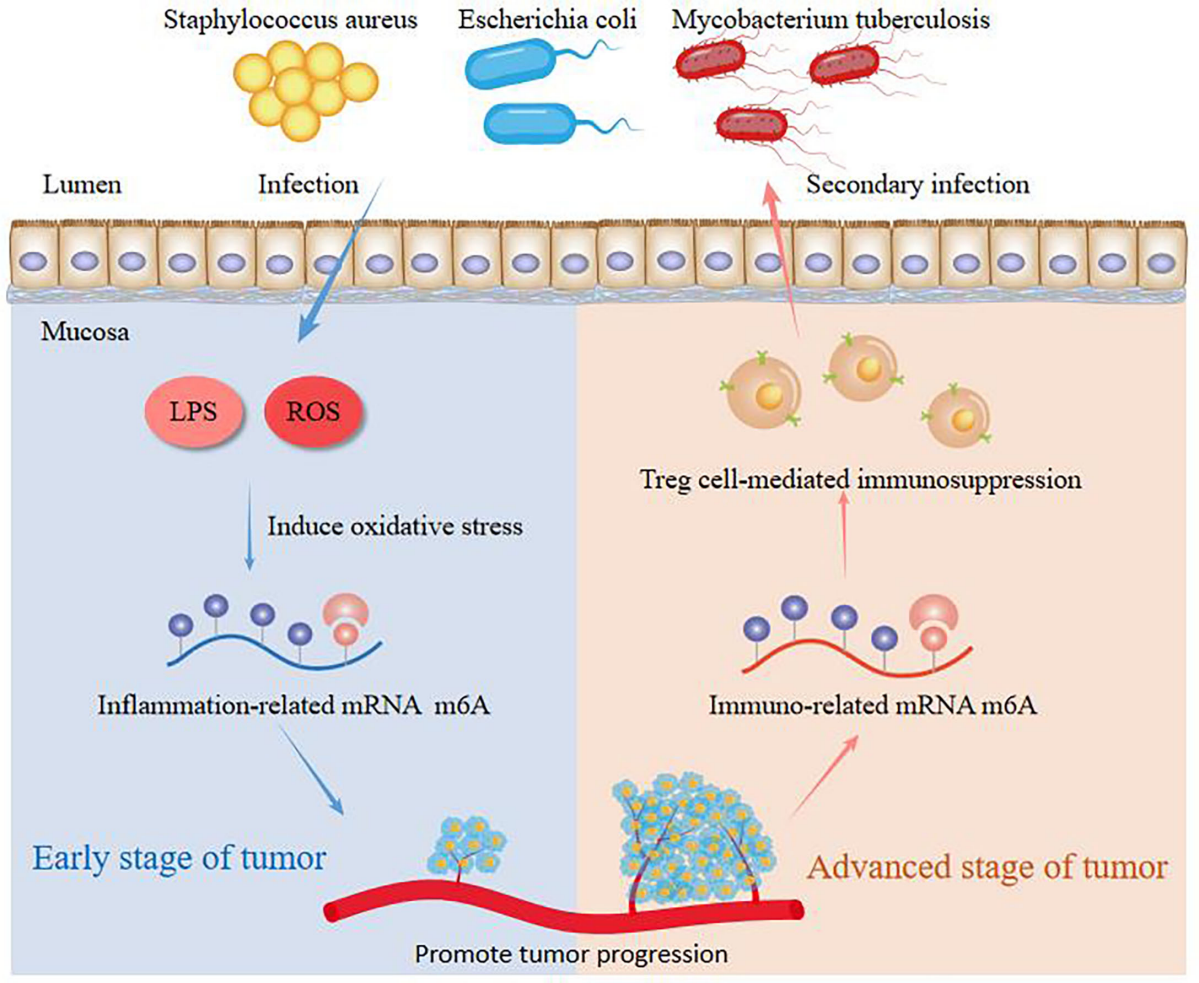
The molecular mechanisms of m6A in the interaction of host and microbes in cancer.

As a universally epigenetic modification, m6A modification is closely related to the occurrence and progression of cancer. Furthermore, it has been identified that intestinal bacteria are involved in supplying the methyl donor substances and modulating m6A RNA methylation, indicating that intestinal bacteria may play an essential role in occurrence and progression of cancers through regulating m6A RNA methylation. Luo et al. demonstrated that bioactive nonstarch polysaccharides dramatically ameliorate cancer by altering host m6A RNA methylation, which influences methyl donors mediated by intestinal microbiota (Luo et al., 2021). They provided us a scientific view that m6A RNA methylation may participate in the occurrence and progression of various types of cancer, including NSCLC, through intestinal microbiota. Additionally, Chen et al. found that Fusobacterium nucleatum significantly reduces m6A modifications in colorectal cancer cells and patient-derived xenograft (PDX) tissues through downregulation of METTL3, leading to enhancement of colorectal cancer aggressiveness (Chen et al., 2022). On the one hand, it was worth noting that lipopolysaccharide (LPS) simulation or reactive oxygen species (ROS) release promotes inflammation-related hepatocellular carcinoma progression in early stage of tumor and is related to unfavorable prognosis through increasing m6A methylation of GNAS mRNA (Ding et al., 2020). Therefore, the pathogen bacteria produce ROS or release LPS to induce oxidative stress to change m6A modification in host, causing poor response to chemotherapy and immunotherapy. On the other hand, Treg cell-mediated immunosuppression in patients at advanced stage of tumor can lead to secondary infection by some opportunistic pathogens like Staphylococcus aureus, Escherichia coli and Mycobacterium tuberculosis in a m6A-dependent manner (**Figure 3**). What’s more, the secondary infection may activate TLR and TLR-mediated tumor-promoting responses, which promote the occurrence and development of lung cancer. Consequently, the above results indicated us that m6A modification may participate in the progression of virous types of cancer by microbiota and similar pattern of regulation probably existed in NSCLC. Nevertheless, the interactions between the commensal microbiota and m6A methylation remain incompletely understood and need further explored.

## Conclusions and prospects

In recent years, the enzymes related to m6A methylation modification, the role and biological significance of m6A in mRNA modification, and the regulatory mechanism of m6A in malignant tumors have been revealed. m6A is precisely regulated by methylesterases, demethylases and recognition proteins, and is involved in almost every step of mRNA biology from production to degradation, and is involved in regulating the production and biological functions of miRNAs and lncRNAs. m6A plays an important role in the metabolism, drug resistance and metastasis of many malignant tumors, suggesting that m6A modification can be a target for the prevention and treatment of human tumors.

The discovery of inhibitors of m6A-related factors (such as FTO, ALKBH5 and HIF) has helped to achieve oncogene-targeted therapy. So far, several FTO inhibitors have been reported (rhodopsin, meclofenamic acid, IOX3, etc.), most of which are not specific. Meclofenamic acid (MA), one of the selective FTO inhibitors, is a non-steroidal anti-inflammatory drug that competes with the FTO binding site. The FTO inhibitor MA2 (an ethyl ester derivative of MA) prolongs the lifespan of GSC-transplanted mice, implying that m6A modification may be a target for inhibiting tumor progression and reversing resistance to radiotherapy and chemotherapy in glioblastoma. In addition, the metabolite R-2HG caused by isocitrate dehydrogenase (IDH) mutation exerts anti-leukemic effects by inhibiting FTO/m6A/MYC/CEBPA signaling, providing a new target for clinical dosing in the treatment of leukemia. Although many inhibitors targeting m6A demethylases have been identified, there is limited *in vivo* evidence for their effects, and there is still room for extensive exploration of the development of m6A-related protein inhibitors and clinical indication options. Firstly, RNA modifications are spatially and temporally dynamic and tissue-specific, and their regulatory mechanisms require single-base precision high-resolution sequencing and measurement technologies, while the current mainstream antibody capture technologies cannot meet the single- base precision targeting of m6A. Secondly, there is no technology to edit the m6A site of the transcript single gene for specific genes. In addition, although some demethylase inhibitors have been identified and provide new targets for oncology drugs, their actions and specific mechanisms are not yet fully understood and lack specificity. Therefore, researchers expect to obtain more inhibitors targeting m6A-associated proteins, especially more specific ones, to provide a new strategy for epigenetic-based tumor targeting therapy.

Several new drugs targeting DNA methylesterases or histone modifying enzymes have been approved for the treatment of tumors with promising therapeutic effects and significant economic benefits. Epigenetic-based chemical interventions have become an active area of research for new drug targets in the international arena. Due to the late start, RNA epigenetic research with m6A modification as the core is still in the early stage. Since targeting the RNA epigenome, including its editing, degradation, translocation and translation, has good advantages in terms of safety and timeliness, the discovery of high-quality chemical probes and specific inhibitors, the development of single-gene specific editing technology, and the targeted intervention of m6A modifications can not only promote basic research in related fields, but also show great prospects for application in tumor therapy and other disease-related fields, and be used in both life science and new drug discovery. What’s more, it has been identified that m6A methylation plays a crucial role in microbiota homeostasis for maintaining physiological balance and stabilization of body, which probably server as a messenger to participate in the crosstalk of host and microbiomes. For one thing, the change of microbiota induced by the m6A may participate in reshaping the microenvironment. For another, microbiota play an essential role in regulating host m6A RNA modification profiles through microbial metabolites, inducing ROS or releasing LPS. More importantly, m6A-related therapeutic strategies provide a promising direction for the targeted therapy in various diseases, especially in tumors.

Therefore, the development of single gene specific editing technologies to target m6A modifications will not only promote basic research in related fields, but also show great promise for applications in disease related fields such as tumor therapy, and show scientific importance in both life science and new drug discovery. These findings not only provide novel mechanistic insight into the biology of lung cancer but also shed light on new therapeutic targets and strategies for lung cancer prevention and treatment.

## Author contributions

F-SQ and Y-SZ searched the articles and drafted the manuscript. M-YG checked the contents. J-QH and C-HY revised the manuscript and provided the funds. C-HY was responsible for the project administration. All authors contributed to the article and approved the submitted version.

## Funding

This work is supported by National Natural Science Foundation of China (No. 82104526), Zhejiang province of medical science (No. 2022KY917), Medical Science and Technology Project of Zhejiang Province (No. 2019339594), Special Fund of Zhejiang Academy of Medical Science (No. 2016Y), Basic and Public Project of Zhejiang Province (No. LGD20H160001).

## Conflict of interest

The authors declare that the research was conducted in the absence of any commercial or financial relationships that could be construed as a potential conflict of interest.

## Publisher’s note

All claims expressed in this article are solely those of the authors and do not necessarily represent those of their affiliated organizations, or those of the publisher, the editors and the reviewers. Any product that may be evaluated in this article, or claim that may be made by its manufacturer, is not guaranteed or endorsed by the publisher.

## References

[B1] Aik W. Demetriades M. Hamdan M. K. Bagg E. A. Yeoh K. K. Lejeune C.. (2013). Structural basis for inhibition of the fat mass and obesity associated protein (FTO). J. Med. Chem. 56 (9), 3680–3688. doi: 10.1021/jm400193d 23547775

[B2] Chen B. Ye F. Yu L. Jia G. Huang X. Zhang X.. (2021). Development of cell-active N6-methyladenosine RNA demethylase FTO inhibitor. J. Am. Chem. Soc 134 (43), 17963–17971. doi: 10.1021/ja3064149 23045983

[B3] Chen S. Zhang L. Li M. Zhang Y. Sun M. Wang L.. (2022). Fusobacterium nucleatum reduces METTL3-mediated m6A modification and contributes to colorectal cancer metastasis. Nat. Commun. 13 (1), 1248. doi: 10.1038/s41467-022-28913-5 35273176 PMC8913623

[B4] Chen D. H. Zhang J. G. Wu C. X. Li Q. (2021). Non-coding RNA m6A modification in cancer: Mechanisms and therapeutic targets. Front. Cell Dev. Biol. 9, 778582. doi: 10.3389/fcell.2021.778582 35004679 PMC8728017

[B5] Choe J. Lin S. Zhang W. Liu Q. Wang L. Ramirez-Moya J.. (2018). mRNA circularization by METTL3-eIF3h enhances translation and promotes oncogenesis. Nature 561 (7724), 556–560. doi: 10.1038/s41586-018-0538-8 30232453 PMC6234840

[B6] Deng L. J. Deng W. Q. Fan S. R. Chen M. F. Qi M. Lyu W. Y.. (2022). m6A modification: recent advances, anticancer targeted drug discovery and beyond. Mol. Cancer 21 (1), 52. doi: 10.1186/s12943-022-01510-2 35164788 PMC8842557

[B7] Desnoyers G. Frost L. D. Courteau L. Wall M. L. Lewis S. M. (2015). Decreased eIF3e expression can mediate epithelial-to-Mesenchymal transition through activation of the TGFβ signaling pathway. Mol. Cancer Res. 13 (10), 1421–1430. doi: 10.1158/1541-7786.MCR-14-0645 26056130

[B8] Desrosiers R. Friderici K. Rottman F. (1974). Identification of methylated nucleosides in messenger RNA from novikoff hepatoma cells. Proc. Natl. Acad. Sci. U.S.A. 71 (10), 3971–3975. doi: 10.1073/pnas.71.10.3971 4372599 PMC434308

[B9] Ding H. Zhang X. Su Y. Jia C. Dai. C. (2020). GNAS promotes inflammation-related hepatocellular carcinoma progression by promoting STAT3 activation. Cell Mol. Biol. Lett. 25, 8. doi: 10.1186/s11658-020-00204-1 32123532 PMC7038622

[B10] Du Y. Hou G. Zhang H. Dou J. He J. Guo Y.. (2018). SUMOylation of the m6A-RNA methyltransferase METTL3 modulates its function. Nucleic Acids Res. 46 (10), 5195–5208. doi: 10.1093/nar/gky156 29506078 PMC6007514

[B11] Esteves P. Dard L. Brillac A. Hubert C. Sarlak S. Rousseau B.. (2020). Nuclear control of lung cancer cells migration, invasion and bioenergetics by eukaryotic translation initiation factor 3F. Oncogene. 39 (3), 617–636. doi: 10.1038/s41388-019-1009-x 31527668 PMC6962096

[B12] Fang C. Chen Y. X. Wu N. Y. Yin J. Y. Li X. P. Huang H. S.. (2017). MiR-488 inhibits proliferation and cisplatin sensibility in non-small-cell lung cancer (NSCLC) cells by activating the eIF3a-mediated NER signaling pathway. Sci. Rep. 7, 40384. doi: 10.1038/srep40384 28074905 PMC5225486

[B13] Gao R. Ye M. Liu B. Wei M. Ma D. Dong K. (2021). m6A modification: A double-edged sword in tumor development. Front. Oncol. 11, 679367. doi: 10.3389/fonc.2021.679367 34381710 PMC8350482

[B14] Han X. Wang N. Li J. Wang Y. Wang R. Chang. J. (2019). Identification of nafamostat mesilate as an inhibitor of the fat mass and obesity-associated protein (FTO) demethylase activity. Chem. Biol. Interact. 297, 80–84. doi: 10.1016/j.cbi.2018.10.023 30393114

[B15] He J.-Q. Chen Q. Wu S.-J. Wang D.-Q. Zhang S.-Y. Zhang S.-Z.. (2022). Potential implications of the lung microbiota in patients with chronic obstruction pulmonary disease and non-small cell lung cancer. Front. Cell. Infect Microbiol. 12. doi: 10.3389/fcimb.2022.937864 PMC9363884 35967848

[B16] He W. Zhou B. Liu W. Zhang M. Shen Z. Han Z.. (2015). Identification of a novel small-molecule binding site of the fat mass and obesity associated protein (FTO). J. Med. Chem. 58 (18), 7341–7348. doi: 10.1021/acs.jmedchem.5b00702 26314339

[B17] Huang Y. Su R. Sheng Y. Dong L. Dong Z. Xu H.. (2019). Small-molecule targeting of oncogenic FTO demethylase in acute myeloid leukemia. Cancer Cell 35 (4), 677–691.e10. doi: 10.1016/j.ccell.2019.03.006 30991027 PMC6812656

[B18] Huang H. Wang Y. Kandpal M. Zhao G. Cardenas H. Ji Y.. (2020). FTO-dependent n 6-methyladenosine modifications inhibit ovarian cancer stem cell self-renewal by blocking cAMP signaling. Cancer Res. 80 (16), 3200–3214. doi: 10.1158/0008-5472.CAN-19-4044 32606006 PMC7442742

[B19] Huang H. Weng H. Sun W. Qin X. Shi H. Wu H.. (2018). Recognition of RNA N6-methyladenosine by IGF2BP proteins enhances mRNA stability and translation. Nat. Cell Biol. 20 (3), 285–295. doi: 10.1038/s41556-018-0045-z 29476152 PMC5826585

[B20] Huang Y. Yan J. Li Q. Li J. Gong S. Zhou H.. (2015). Meclofenamic acid selectively inhibits FTO demethylation of m6A over ALKBH5. Nucleic Acids Res. 43 (1), 373–384. doi: 10.1093/nar/gku1276 25452335 PMC4288171

[B21] Hu J. Qiu D. Yu A. Hu J. Deng H. Li H.. (2021). YTHDF1 is a potential pan-cancer biomarker for prognosis and immunotherapy. Front. Oncol. 11, 607224. doi: 10.3389/fonc.2021.607224 34026603 PMC8134747

[B22] Jin L. Chen C. Yao J. Yu Z. Bu L. (2022). The RNA N^6^-methyladenosine modulator HNRNPA2B1 is involved in the development of non-small cell lung cancer. Clin. Exp. Pharmacol. Physiol. 49 (3), 329–340. doi: 10.1111/1440-1681.13608 34717005

[B23] Jin D. Guo J. Wu Y. Du J. Yang L. Wang X.. (2019). m6A mRNA methylation initiated by METTL3 directly promotes YAP translation and increases YAP activity by regulating the MALAT1-miR-1914-3p-YAP axis to induce NSCLC drug resistance and metastasis. J. Hematol. Oncol. 12 (1), 135. doi: 10.1186/s13045-019-0830-6 31818312 PMC6902496

[B24] Jin D. Guo J. Wu Y. Du J. Yang L. Wang X.. (2021). m6A mRNA methylation initiated by METTL3 directly promotes YAP translation and increases YAP activity by regulating the MALAT1-miR-1914-3p-YAP axis to induce NSCLC drug resistance and metastasis. J. Hematol. Oncol. 14 (1), 32. doi: 10.1186/s13045-021-01048-8 33618740 PMC7901070

[B25] Jin D. Guo J. Wu Y. Yang L. Wang X. Du J.. (2020). m6A demethylase ALKBH5 inhibits tumor growth and metastasis by reducing YTHDFs-mediated YAP expression and inhibiting miR-107/LATS2-mediated YAP activity in NSCLC. Mol. Cancer 19 (1), 40. doi: 10.1186/s12943-020-01161-1 32106857 PMC7045432

[B26] Kim M. K. Choi M. J. Lee H. M. Choi H. S. Park Y. K. Ryu C. J. (2021). Heterogeneous nuclear ribonucleoprotein A2/B1 regulates the ERK and p53/HDM2 signaling pathways to promote the survival, proliferation and migration of non−small cell lung cancer cells. Oncol. Rep. 46 (2), 153. doi: 10.3892/or.2021.8104 34109989

[B27] Kumari R. Ranjan P. Suleiman Z. G. Goswami S. K. Li J. Prasad R.. (2021). mRNA modifications in cardiovascular biology and disease: with a focus on m6A modification. Cardiovasc. Res. 118 (7), 1680–1692. doi: 10.1093/cvr/cvab160 PMC9630866 33956076

[B28] Li J. Han Y. Zhang H. Qian Z. Jia W. Gao Y.. (2019). The m6A demethylase FTO promotes the growth of lung cancer cells by regulating the m6A level of USP7 mRNA. Biochem. Biophys. Res. Commun. 512 (3), 479–485. doi: 10.1016/j.bbrc.2019.03.093 30905413

[B29] Lin S. Choe J. Du P. Triboulet R. Gregory R. I. (2016). The m(6)A methyltransferase METTL3 promotes translation in human cancer cells. Mol. Cell. 62 (3), 335–345. doi: 10.1016/j.molcel.2016.03.021 27117702 PMC4860043

[B30] Lin Z. Xiong L. Lin Q. (2015). Knockdown of eIF3d inhibits cell proliferation through G2/M phase arrest in non-small cell lung cancer. Med. Oncol. 32 (7), 183. doi: 10.1007/s12032-015-0625-8 26008152

[B31] Liu J. Ren D. Du Z. Wang H. Zhang H. Jin Y. (2018). m6A demethylase FTO facilitates tumor progression in lung squamous cell carcinoma by regulating MZF1 expression. Biochem. Biophys. Res. Commun. 502 (4), 456–464. doi: 10.1016/j.bbrc.2018.05.175 29842885

[B32] Li J. Wang Y. Han X. Wang N. Yu W. Wang R.. (2019). The role of chlorine atom on the binding between 2-phenyl-1H-benzimidazole analogues and fat mass and obesity-associated protein. J. Mol. Recognit. 32 (6), e2774. doi: 10.1002/jmr.2774 30575149

[B33] Luo J. Yu J. Peng X. (2021). Could partial nonstarch polysaccharides ameliorate cancer by altering m6A RNA methylation in hosts through intestinal microbiota? Crit. Rev. Food Sci. Nutr. 26, 1–16. doi: 10.1080/10408398.2021.1927975 34036843

[B34] Pei Y. Lou X. Li K. Xu X. Guo Y. Xu D.. (2020). Peripheral blood leukocyte N6-methyladenosine is a noninvasive biomarker for non-small-cell lung carcinoma. OncoTargets Ther. 13, 11913–11921. doi: 10.2147/ott.S267344 PMC7682600 33239892

[B35] Peng S. Xiao W. Ju D. Sun B. Hou N. Liu Q.. (2019). Identification of entacapone as a chemical inhibitor of FTO mediating metabolic regulation through FOXO1. Sci. Transl. Med. 11 (488), eaau7116. doi: 10.1126/scitranslmed.aau7116 30996080

[B36] Prakash M. Itoh Y. Fujiwara Y. Takahashi Y. Takada Y. Mellini P.. (2021). Identification of potent and selective inhibitors of fat mass obesity-associated protein using a fragment-merging approach. J. Med. Chem. 64 (21), 15810–15824. doi: 10.1021/acs.jmedchem.1c01107 34727689

[B37] Qiao Y. Zhou B. Zhang M. Liu W. Han Z. Song C.. (2016). A novel inhibitor of the obesity-related protein FTO. Biochemistry 55 (10), 1516–1522. doi: 10.1021/acs.biochem.6b00023 26915401

[B38] Shen J. Yin J. Y. Li X. P. Liu Z. Q. Wang Y. Chen J.. (2014). The prognostic value of altered eIF3a and its association with p27 in non-small cell lung cancers. PloS One 9 (4), e96008. doi: 10.1371/journal.pone.0096008 24789280 PMC4005749

[B39] Shi Y. Fan S. Wu M. Zuo Z. Li X. Jiang L.. (2019). YTHDF1 links hypoxia adaptation and non-small cell lung cancer progression. Nat. Commun. 10 (1), 4892. doi: 10.1038/s41467-019-12801-6 31653849 PMC6814821

[B40] Shi L. Gong Y. Zhuo L. Wang S. Chen S. Ke B. (2022). Methyltransferase-like 3 upregulation is involved in the chemoresistance of non-small cell lung cancer. Ann. Trans. Med. 10 (3), 139. doi: 10.21037/atm-21-6608 PMC8904991 35284536

[B41] Shi C. Y. Yu C. H. Yu W. Y. Ying. H. Z. (2021). Gut-lung microbiota in chronic pulmonary diseases: Evolution, pathogenesis, and therapeutics. Can. J. Infect. Dis. Med. Microbiol. 2021, 9278441. doi: 10.1155/2021/9278441 34900069 PMC8664551

[B42] Tang Y. Chen K. Song B. Ma J. Wu X. Xu Q.. (2021). m6A-atlas: a comprehensive knowledgebase for unraveling the N6-methyladenosine (m6A) epitranscriptome. Nucleic Acids Res. 49 (D1), D134–D143. doi: 10.1093/nar/gkaa692 32821938 PMC7779050

[B43] Wang X. Feng J. Xue Y. Guan Z. Zhang D. Liu Z.. (2016). Structural basis of N(6)-adenosine methylation by the METTL3-METTL14 complex. Nature 534 (7608), 575–578. doi: 10.1038/nature18298 27281194

[B44] Wang R. Han Z. Liu B. Zhou B. Wang N. Jiang Q.. (2018). Identification of natural compound radicicol as a potent FTO inhibitor. Mol. Pharm. 15 (9), 4092–4098. doi: 10.1021/acs.molpharmaceut.8b00522 30063141

[B45] Wang Y. Li J. Han X. Wang N. Song C. Wang R.. (2019). Identification of clausine e as an inhibitor of fat mass and obesity-associated protein (FTO) demethylase activity. J. Mol. Recognit. 32 (10), e2800. doi: 10.1002/jmr.2800 31321808

[B46] Wang Y. Li M. Zhang L. Chen Y. Zhang S. (2021). m6A demethylase FTO induces NELL2 expression by inhibiting E2F1 m6A modification leading to metastasis of non-small cell lung cancer. Mol. Ther. Oncolytics 21, 367–376. doi: 10.1016/j.omto.2021.04.011 34169146 PMC8190133

[B47] Wang J. Wang J. Gu Q. Ma Y. Yang Y. Zhu J.. (2020). The biological function of m6A demethylase ALKBH5 and its role in human disease. Cancer Cell Int. 20, 347. doi: 10.1186/s12935-020-01450-1 32742194 PMC7388453

[B48] Xie L. J. Wang R. L. Wang D. Liu L. Cheng L. (2017). Visible-light-mediated oxidative demethylation of N6-methyl adenines. Chem. Commun. (Camb). 53 (77), 10734–10737. doi: 10.1039/c7cc05544g 28920612

[B49] Xu X. Huang J. Ocansey D. K. W. Xia Y. Zhao Z. Xu Z.. (2021). The emerging clinical application of m6A RNA modification in inflammatory bowel disease and its associated colorectal cancer. J. Inflammation Res. 14, 3289–3306. doi: 10.2147/JIR.S320449 PMC8289367 34290515

[B50] Xu C. Liu K. Tempel W. Demetriades M. Aik W. Schofield C. J.. (2014). Structures of human ALKBH5 demethylase reveal a unique binding mode for specific single-stranded N6-methyladenosine RNA demethylation. J. Biol. Chem. 289 (25), 17299–17311. doi: 10.1074/jbc.M114.550350 24778178 PMC4067165

[B51] Yang F. Yuan W. Q. Li J. Luo. Y. Q. (2021). Knockdown of METTL14 suppresses the malignant progression of non-small cell lung cancer by reducing twist expression. Oncol. Lett. 22 (6), 847. doi: 10.3892/ol.2021.13108 34733365 PMC8561617

[B52] Yan M. Sun L. Li J. Yu H. Lin H. Yu T.. (2019). RNA-Binding protein KHSRP promotes tumor growth and metastasis in non-small cell lung cancer. J. Exp. Clin. Cancer Res. 38 (1), 478. doi: 10.1186/s13046-019-1479-2 31775888 PMC6882349

[B53] Yin Y. Long J. Sun Y. Li H. Jiang E. Zeng C.. (2018). The function and clinical significance of eIF3 in cancer. Gene. 673, 130–133. doi: 10.1016/j.gene.2018.06.034 29908282

[B54] Zaccara S. Ries R. J. Jaffrey S. R. (2019). Writing and erasing mRNA methylation. Nat. Rev. Mol. Cell Biol. 20 (10), 608–624. doi: 10.1038/s41580-019-0168-5 31520073

[B55] Zeng C. Huang W. Li Y. Weng H. (2020). Roles of METTL3 in cancer: mechanisms and therapeutic targeting. J. Hematol. Oncol. 13 (1), 117. doi: 10.1186/s13045-020-00951-w 32854717 PMC7457244

[B56] Zhang Y. Liu S. Zhao T. Dang C. (2021). METTL3−mediated m6A modification of Bcl−2 mRNA promotes non−small cell lung cancer progression. Oncol. Rep. 46 (2), 163. doi: 10.3892/or.2021.8114 34132367 PMC8218297

[B57] Zhang H. Shi X. Huang T. Zhao X. Chen W. Gu N.. (2020). Dynamic landscape and evolution of m6A methylation in human. Nucleic Acids Res. 48 (11), 6251–6264. doi: 10.1093/nar/gkaa347 32406913 PMC7293016

[B58] Zheng G. Dahl J. A. Niu Y. Fedorcsak P. Huang C. M. Li C. J.. (2013). ALKBH5 is a mammalian RNA demethylase that impacts RNA metabolism and mouse fertility. Mol. Cell 49 (1), 18–29. doi: 10.1016/j.molcel.2012.10.015 23177736 PMC3646334

[B59] Zhou J. Xiao D. Qiu T. Li J. Liu Z. (2020). Loading MicroRNA-376c in extracellular vesicles inhibits properties of non-small cell lung cancer cells by targeting YTHDF1. Technol. Cancer Res. Treat 19, 1533033820977525. doi: 10.1177/153303382097752 33280517 PMC7724269

